# Characterization of the gut micro biota in Koreans and investigation of its association with probiotic consumption: implications for microbial ecology and host health

**DOI:** 10.3389/fmicb.2025.1745533

**Published:** 2026-01-30

**Authors:** Yo-Ram Uh, Si-Nae Park, Min-Jung Song

**Affiliations:** Department of BT Research Institute, U2 Bio Co. Ltd., Seoul, Republic of Korea

**Keywords:** age-related changes, gut micro biota, Korean cohort, microbial diversity, probiotic supplementation

## Abstract

**Introduction:**

The gut micro biota is reportedly closely related to human health, and its composition and diversity are determined by a variety of factors, including age, diet, and probiotic intake. Although many studies on the gut micro biota have been conducted, most have focused on Western populations or have been limited by small sample sizes, making it difficult to understand micro biota differences across populations and lifestyles. In this study, we analyzed a large Korean cohort of 3,450 individuals, focusing on gut micro biome differences according to age and host-related markers, as well as the impact of probiotic supplementation.

**Methods:**

Fecal samples from 3,450 Koreans were analyzed using 16S rRNA gene sequencing (V3–V4 region). Bioinformatics and taxonomic analyses were performed to compare microbial composition and diversity according to age and probiotic intake.

**Results:**

The data revealed a significant increase in microbial diversity with age and distinct shifts in taxonomic composition between younger and older participants. In addition, probiotic intake did not alter overall community diversity but increased the detection of probiotics, suggesting that they serve as moderators rather than direct drivers of diversity.

**Conclusion:**

These findings emphasize the importance of population-specific micro biome research and suggest that diverse host-related and lifestyle factors jointly contribute to shaping gut microbial ecology in Koreans. Probiotic supplementation primarily increased the detection of specific lactic acid bacteria and bifidobacterial species without substantially altering overall alpha diversity, consistent with a modulatory role on targeted taxa rather than broad community restructuring. Together, these results provide a useful framework for future studies linking probiotic-responsive microbial features to human health outcomes and for developing precision nutrition and probiotic strategies in Korean and similar populations.

## 1 Introduction

The gut micro biota, composed of trillions of microorganisms, has been recognized as a central determinant of human health. These microbial communities play essential roles in nutrient metabolism, immune regulation, and defense against pathogens, while maintaining close connections with neurological and metabolic functions (Fan and Pedersen, 2021; Lynch and Pedersen, 2016; Nicholson et al., 2012). Disruptions in microbial homeostasis have been linked to a wide range of conditions, including obesity, type 2 diabetes, inflammatory bowel disease, and certain cancers (Cani, 2018; Shreiner et al., 2015; Zmora et al., 2019). Therefore, understanding the factors that govern the composition and diversity of the gut micro biota remains a primary focus in micro biome research. Accordingly, a central challenge is to understand how intrinsic host factors and modifiable lifestyle exposures jointly shape gut microbial communities within specific populations.

Among the factors influencing gut micro biota, age has consistently been highlighted as a key determinant of microbial composition. Previous studies have demonstrated that microbial diversity tends to increase with age, accompanied by shifts in the relative abundance of specific bacterial taxa across life stages (Odamaki et al., 2016; Xu et al., 2019). However, these trajectories appear to be shaped by dietary patterns, lifestyle, genetic predispositions, and cultural background, leading to inconsistent findings across populations (Parizadeh and Arrieta, 2023; van der Vossen et al., 2023; Yatsunenko et al., 2012). Further, research examining age-related microbial variation in East Asian populations remains limited, underscoring the need to investigate these patterns in specific regional and cultural contexts.

In this context, Korea is characterized by a unique dietary culture rich in fermented foods, a progressively urbanized lifestyle, and increasing consumption of probiotics—factors that are expected to jointly influence gut microbial dynamics. Middle age in Korea—typically beginning in the 40s—represents an important turning point in health status, during which substantial changes in metabolic risk factors and lifestyle patterns occur. Epidemiological data from Korean adults indicate that the prevalence of metabolic syndrome and related cardiometabolic risk factors sharply increase from the 40s onward, with men in midlife showing particularly elevated risk compared with younger age groups (Huh et al., 2021; Kwon et al., 2005). In parallel, national health surveys have documented worsening trends in obesity, insufficient leisure-time physical activity, and sedentary behavior among Korean adults, with midlife groups contributing substantially to the growing cardiometabolic burden (Han et al., 2024). Taken together, these findings suggest that, from the 40s onward, accumulated lifestyle changes—such as reduced physical activity, increased sedentary time, and other shifts in health behaviors—begin to manifest as increased metabolic risk within this distinct dietary and cultural environment.

Probiotics, defined as live microorganisms that confer health benefits to the host when consumed in adequate amounts, are widely used to promote gut health (Guarner et al., 2024). Previous studies have shown that probiotic intake can promote the growth of beneficial genera such as *Bifidobacterium* and *Lactobacillus* and provide functional advantages to the broader microbial community (Ejima et al., 2024; Hemarajata and Versalovic, 2013). However, large-scale, population-based studies that directly compare species-level detection rates before and after probiotic use remain scarce. Moreover, it is still unclear how demographic and lifestyle factors—including sex, body mass index (BMI), and smoking status—shape probiotic-associated microbial patterns in Korean populations. In this setting, where studies disentangling the relationships among metabolic syndrome, lifestyle factors, and gut microbial dysbiosis are still limited, understanding how intrinsic determinants (e.g., age) and modifiable lifestyle variables (e.g., smoking status, BMI, probiotic intake) influence the gut micro biota of Koreans is essential for developing population-tailored insights that can support precision nutrition and microbial health strategies.

Against this background, the overarching aim of the present study was to identify the major host determinants that shape the gut micro biota of Koreans in a large real-world population. Using a cohort of 3,450 Korean adults, we sought to: (i) characterize age-related differences in alpha diversity and microbial composition, with particular attention to the midlife transition around 40 years; (ii) investigate how demographic and lifestyle factors, including sex, BMI, smoking status, and probiotic intake, are associated with the detection of probiotic taxa; and (iii) assess within-individual changes in probiotic species detection before and after probiotic use. In addition, we performed an exploratory comparison with a smaller Thai cohort to provide a preliminary context for cross-population differences. Through these integrated analyses, this study provides foundational data on microbial ecology in Koreans while offering insight into host- and lifestyle-dependent mechanisms of gut micro biota variation that are likely to be relevant across human populations.

## 2 Materials and methods

### 2.1 Study participants and data collection

Data were collected from a total of 3,450 Korean participants who underwent gut micro biota testing through the U2biome service (U2bio, Seoul, Republic of Korea) between March 2021 and October 2024. The structured survey included demographic information (age and sex), health-related variables (BMI and smoking status), and probiotic intake history. Probiotic intake was assessed using a structured questionnaire that asked whether participants were currently taking any probiotic products (yes/no); information on specific brands, strains, doses, formulations (single-strain vs. multi-strain), and duration or frequency of use was not collected. Participants were classified as non-obese (BMI < 23) or obese (BMI ≥ 23), based on World Health Organization guidelines (World Health Organization, 2000).

Among the total participants, a subgroup of 558 individuals underwent microbiome testing both before and after probiotic intake. This subgroup was used to evaluate changes in the detection of probiotic strains following supplementation, based on self-reported initiation of probiotic use between the two sampling time points. The 19 probiotic species approved by the Ministry of Food and Drug Safety (MFDS), Republic of Korea, were used for the analysis (Supplementary Table 1). All personally identifying information, such as names and dates of birth, was anonymized prior to analysis. For **cross**-national comparison, an exploratory analysis was conducted using data from 73 Thai individuals recruited via the U2Thai in Bangkok and 73 Korean individuals who underwent testing during the same period. Personally identifiable information in this subgroup was fully anonymized, and all data were de-identified before analysis. The requirement for written informed consent was formally waived by the Institutional Review Board of U2 Medical Foundation (IRB No. 2024045_MU01).

### 2.2 Stool sample collection and DNA extraction

Microbial genomic DNA was extracted from stool samples using the QIAamp PowerFecal Pro DNA Kit (Qiagen, Germany), following the manufacturer's instructions. The concentration and purity of the extracted DNA were assessed using both a NanoDrop spectrophotometer and a Qubit fluorometer (Thermo Fisher Scientific, USA).

### 2.3 16S rRNA gene amplification and sequencing

Microbial community profiling was performed by targeting the V3–V4 hypervariable regions of the 16S rRNA gene using the 16S Metagenomic Sequencing Library Preparation protocol (Illumina, San Diego, CA, USA). PCR amplification was carried out using the primers 341F (5′-TCGTCGGCAGCGTCAGATGTGTATAAGAGACAG-CCTACGGGNGGCWGCAG-3′) and 805R (5′-GTCTCGTGGGCTCGGAGATGTGTATAAGAGACAG-GACTACHVGGGTATCTAATCC-3′). The thermal cycling conditions for the primary PCR were as follows: initial denaturation at 95 °C for 3 min; 25 cycles of denaturation at 95 °C for 30 s, annealing at 55 °C for 30 s, and extension at 72 °C for 30 s; followed by a final extension at 72 °C for 5 min. A secondary PCR was conducted to attach Illumina Nextera indices (IDT Nextera-compatible 8 bp index in 1_192 format), under the same cycling conditions but for 8 cycles. The final libraries were quantified and sized using a Qubit 4 Fluorometer (Invitrogen, USA) and TapeStation 4200 system (Agilent, USA). Samples that met quality criteria based on the manufacturer's guidelines were subjected to sequencing using the MiSeq Reagent Kit v2 (500-cycles) (Cat. MS-102-2003, Illumina, USA).

### 2.4 Bioinformatics and taxonomic analysis

Sequencing data in FASTQ format were analyzed using the Quantitative Insights Into Microbial Ecology 2 (QIIME2) pipeline. As the quality scores in this study were encoded in Phred33, quality filtering was performed after demultiplexing to evaluate the sequence quality. For phylogenetic diversity analysis, a phylogenetic tree was constructed, and alpha diversity, which reflects within-sample microbial diversity, was assessed after rarefying each sample to a minimum sequencing depth of 20,000 reads. A rarefaction depth of 20,000 reads was selected based on the sequencing depth distribution across samples to ensure sufficient coverage for diversity analysis. Three alpha diversity indices—Shannon's diversity index, Faith's phylogenetic diversity (Faith-pd), and Chao1—were calculated using QIIME2′s standard analytical procedures. Finally, taxonomic classification was performed to determine the microbial composition of each sample. Initial taxonomy was assigned using the Greengenes 13_8 reference database within our established QIIME2-based pipeline to maintain continuity with previous and ongoing analyses in this cohort. To improve species-level resolution, representative 16S rRNA gene sequences were additionally compared against curated NCBI RefSeq reference sequences, and species-level labels were retained only when supported by high-identity matches (>99.7%); otherwise, assignments were kept at the genus or higher taxonomic levels.

### 2.5 Statistical analysis

Statistical comparisons of alpha diversity indices between two independent groups were performed using a two-tailed Student's t-test. For within-subject comparisons of probiotic detection before and after probiotic intake, a paired *t*-test was used. The chi-squared test was applied to compare categorical variables, such as the proportion of individuals with three or more detected probiotic species. For the species-level analysis, detection of each of the 19 MFDS-listed probiotic species was coded as a binary variable (detected vs. not detected), and detection rates were compared between pre- and post-intake samples using chi-squared or Fisher's exact tests, as appropriate. *P*-values from these species-level comparisons were adjusted for multiple testing using the Benjamini–Hochberg false discovery rate (FDR) procedure. For figures presenting proportions, 95% confidence intervals (CIs) were calculated and displayed as error bars where indicated.

To evaluate the associations between host factors and probiotic species detection, multivariable logistic regression models were fitted separately for each probiotic species, with sex, BMI category, and smoking status included as predictors and species detection (present vs. absent) as the outcome. In preliminary models, species with very low detection counts frequently exhibited quasi- or complete separation, non-convergence, and extremely wide CIs, indicating unstable parameter estimates. Therefore, multivariable models were restricted to species detected in at least 20 participants, and species below this prevalence threshold were not modeled. Details of species prevalence and model inclusion are provided in the Results and Supplementary Table 2. For the multivariable models, *p*-values for each species–predictor combination were obtained from Wald tests and adjusted for multiple testing using the Benjamini–Hochberg FDR procedure.

Statistical significance was defined as a two-sided *p* < 0.05, and FDR-adjusted *p* < 0.05 was used for multiple-testing corrected analyses. All statistical analyses were conducted using R software (version 4.4.2; R Foundation for Statistical Computing, Vienna, Austria).

## 3 Results

### 3.1 Age-related trends in the gut micro biota of 3,450 Korean individuals

To investigate age-related patterns in the gut micro biota of the 3,450 Korean individuals, two major analyses were conducted: (1) assessment of alpha diversity across age groups and (2) comparison of the most frequently detected bacterial genera by age. The age distribution of the participants was as follows: teenagers *(n* = 57), 20s (*n* = 572), 30s (*n* = 1,161), 40s (*n* = 728), 50s (*n* = 579), 60s (*n* = 265), and ≥ 70 years (i = 89). For statistical comparisons, participants were stratified into two age groups: < 40 years (*n* = 1,790) and ≥40 years (*n* = 1,660). This classification ensured a relatively balanced sample size between groups, enhancing the statistical power and comparability of the analyses.

#### 3.1.1 Age-dependent changes in alpha diversity indices

**Figure 1:**
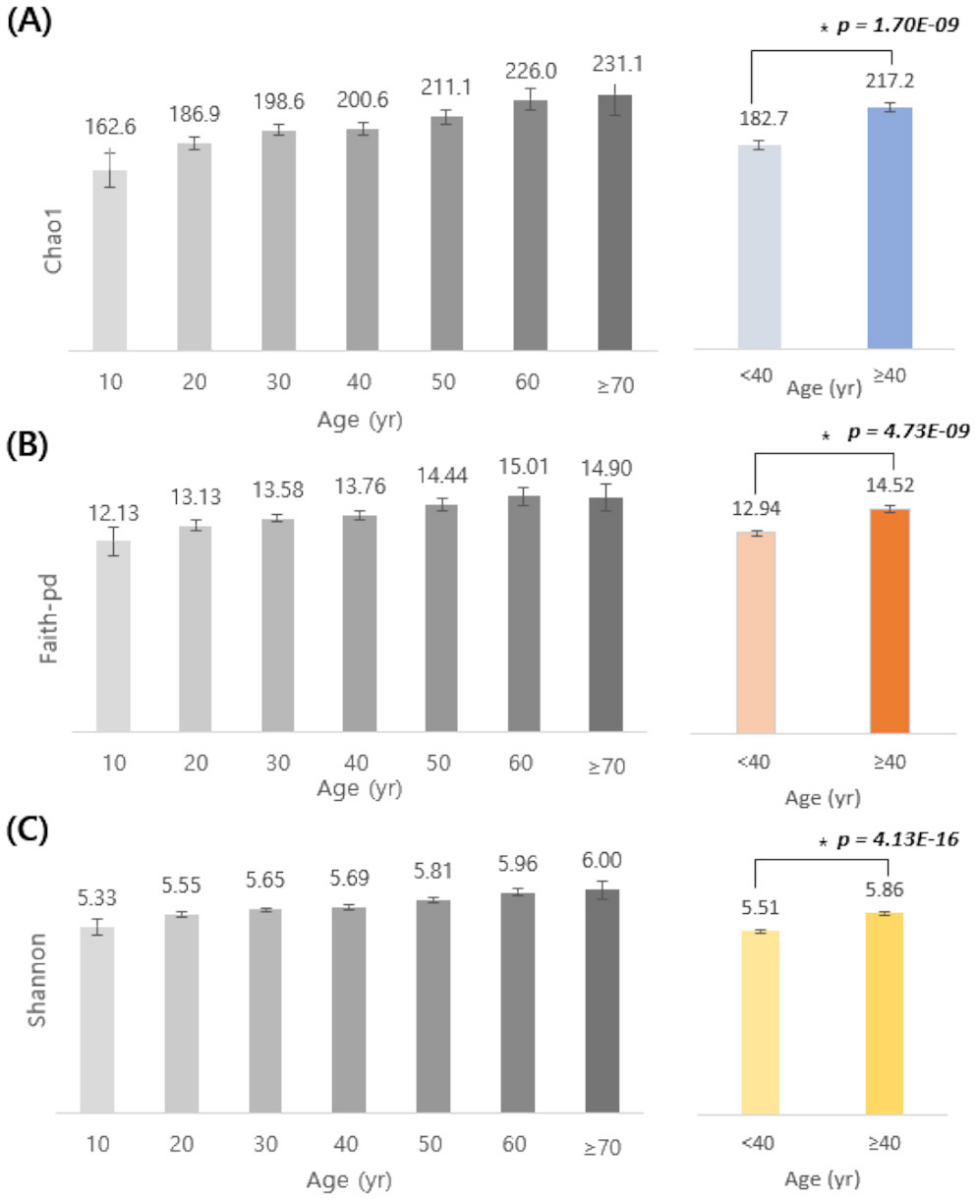
Age-related differences in alpha diversity indices (Chao1, Faith-pd, and Shannon). Alpha diversity indices (**A**, Chao1; **B**, Faith-pd; **C**, Shannon) are shown by age groups (10–70+ years, left) and by two combined groups (<40 yr vs. ≥40 yr, right). Bars indicate mean values and error bars indicate 95% confidence intervals. Statistically significant differences between participants aged <40 and ≥40 years were assessed using a two-sided Student's *t*-test (*Chao1: *p* = 1.70 × 10^−9^; Faith- pd: *p* = 4.73 × 10^−9^; Shannon: *p* = 4.13 × 10^−16^). Colors denote the two age categories (<40 vs. ≥40).

Alpha diversity was assessed across a wide range of age groups (from teens to individuals aged 70 and above) using three indices: Chao1, Faith-pd, and Shannon index (Figure 1). Both the Chao1 and Shannon indices showed a consistent increase with age. Faith-pd followed a similar upward trend but showed a slight decrease in individuals aged 70 and older. To better characterize these trends, participants were stratified into two groups: < 40 and ≥ 40 years. All three indices were significantly higher in the ≥40 group than in the < 40 group (Chao1: 217.2 vs. 182.7; Faith-pd: 14.52 vs. 12.94; Shannon: 5.86 vs. 5.51; *p* < 0.05 by independent *t*-test), indicating that microbial diversity tends to increase with age.

#### 3.1.2 Age-related shifts in the composition of major gut microbial genera

**Figure 2:**
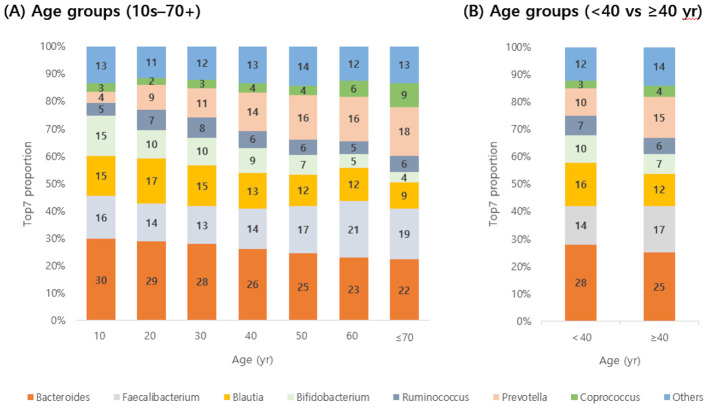
Detection rates of seven predominant gut microbes by age group. Detection rates of the top seven genera (**A**, by decade from 10 to 70+ years; **B**, by combined groups <40 yr and ≥40 yr) are shown. *Bacteroides* decreased with age, whereas *Prevotella* increased.

The composition of gut microbial genera was analyzed across age groups, focusing on the seven most frequently detected genera—*Bacteroides, Faecalibacterium, Blautia, Bifidobacterium, Ruminococcus, Prevotella*, and *Coprococcus*—as well as all other genera grouped together as “Others” (Figure 2). *Bacteroides* was consistently the most dominant genus across all age groups. *Faecalibacterium* ranked second in most groups, except for individuals in their 20s and 30s, where *Blautia* was more predominant. As age increased, the relative abundance of *Bifidobacterium* and *Bacteroides* tended to decrease, while *Coprococcus* and *Prevotella* showed increasing trends. When participants were divided into two groups (<40 and ≥40 years), the proportion of *Bifidobacterium* decreased from 10% to 7%, and that of *Bacteroides* from 28% to 25%. In contrast, that of *Coprococcus* increased from 3% to 4%, and that of *Prevotella* from 10% to 15%.

### 3.2 Associations of probiotic species detection with sex, BMI, and smoking

**Figure 3:**
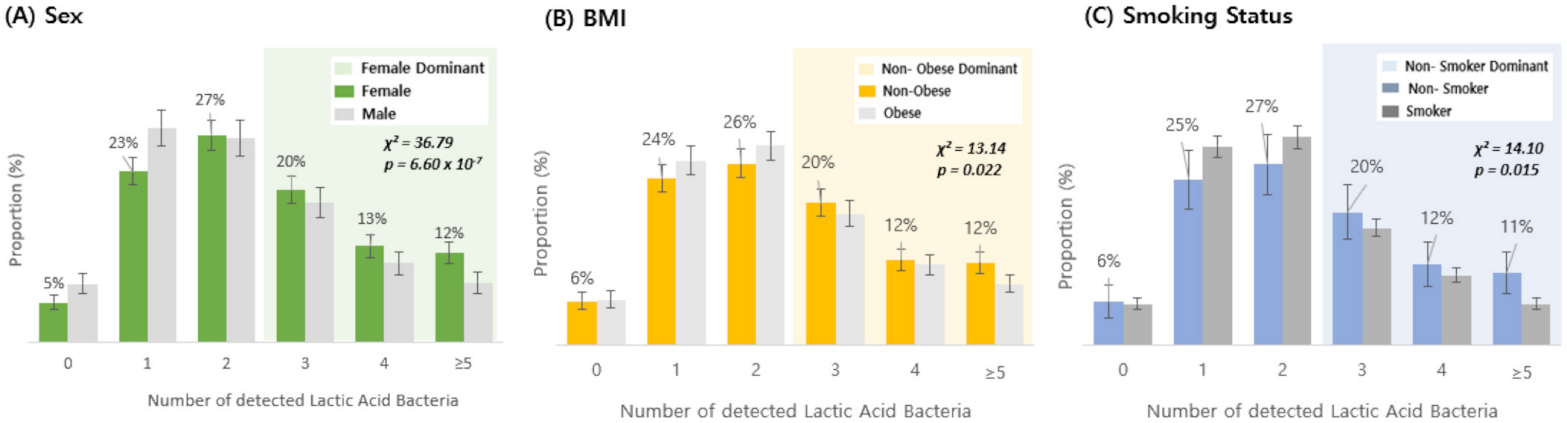
Detection rate of lactic acid bacteria by sex, BMI, and smoking status. The distribution of individuals based on the number of detected lactic acid bacteria (19 species) was compared between groups: **(A)** female vs. male, **(B)** non-obese vs. obese, and **(C)** non-smoker vs. smoker. Shaded areas highlight regions where the dominant group exhibited a higher detection proportion: green shading indicates the range where females had a higher detection proportion than males, yellow shading indicates the range where non-obese individuals had a higher detection proportion than obese individuals, and blue shading indicates the range where non-smokers had a higher detection proportion than smokers. Bars indicate proportions, and error bars represent 95% confidence intervals for the corresponding proportions (normal approximation). Statistical significance was determined by chi-squared test (Sex: χ^2^ = 36.79, *p* = 6.60 × 10^−7^; BMI: χ^2^ = 13.14, *p* = 0.022; smoking status: χ^2^ = 14.10, *p* = 0.015).

Based on questionnaire data from the 3,450 Korean individuals, the number of detected species among 19 types of lactic acid bacteria was analyzed according to gender, BMI, and smoking status (Figure 3). In terms of sex, the proportion of individuals with three or more detected probiotic species was lower in men than in women; similarly, non-obese individuals had a higher proportion of three or more detections than obese individuals, and non-smokers had a higher detection rate of three or more species than smokers (Figures 3A–C). Chi-squared tests revealed statistically significant differences across all three questionnaire variables (*p* < 0.05), and the detailed detection percentages and sample sizes are presented in Table 1. In contrast, alpha diversity indices did not show any statistically significant differences across these host-factor categories. To assess whether the observed associations between host factors and probiotic detection persisted after mutual adjustment, we next fitted multivariable logistic regression models, with sex, BMI category, and smoking status as predictors. Among the 19 targeted probiotic species, 14 met a pre-specified prevalence criterion of at least 20 detected samples and were included in the models; five very low-prevalence species (*L. acidophilus, L. casei, L. paracasei, L. rhamnosus*, and *E. faecium*), each detected in fewer than 20 individuals, could not be modeled reliably and were therefore excluded from the multivariable analyses (Supplementary Table 2). After Benjamini–Hochberg FDR correction across all species–predictor combinations, significant associations were observed for *B. bifidum, B. breve*, and *L. helveticus* (Table 2): male sex was associated with lower odds of detection for *B. bifidum* (OR 0.67, 95% CI 0.57–0.78, FDR = 1.4 × 10^−5^), *B. breve* (OR 0.69, 95% CI 0.55–0.86, FDR = 6.9 × 10^−3^), and *L. helveticus* (OR 0.71, 95% CI 0.57–0.88, FDR = 9.0 × 10^−3^) than female sex, and obesity (vs. non-obesity) was associated with reduced detection of *B. breve* (OR 0.68, 95% CI 0.55–0.84, FDR = 4.0 × 10^−3^). No other associations remained significant after FDR correction, although nominal associations with smoking status were observed for *B. bifidum* and *B. breve* (raw *p* < 0.05 but FDR ≥ 0.05). We reported both raw and FDR-adjusted *p*-values, and associations with FDR-adjusted *p* < 0.05 were considered statistically significant.

**Table 1:** Detection rates of 19 probiotic species by sex, BMI, and smoking status.

**Number of lactic acid bacteria**		**0**	**1**	**2**	**3**	**4**	**≥5**
Sex	Female (*n* = 1,994)	5%	23%	27%	20%	13%	12%
	Male (*n* = 1,456)	8%	28%	27%	19%	10%	8%
BMI	Non-obese (*n* = 1,683)	6%	24%	26%	20%	12%	12%
	Obese (*n* = 1,767)	7%	26%	28%	19%	11%	9%
^*^Smoking status	Non-smoker (*n* = 3,057)	6%	24%	27%	20%	12%	11%
	Smoker (*n* = 380)	6%	30%	31%	17%	10%	6%
Total (*n* = 3,450)		6%	25%	27%	20%	12%	10%

* Smoking status was not reported by 13 individuals.

**Table 2:** Multivariable logistic regression for detection of selected probiotic species.

**Species**	**Predictor**	**OR**	**CI_95**	**p_raw**	**p_FDR**
*B. bifidum*	^*^Sex (Male vs. Female)	0.67	0.57–0.78	0.000000962	0.0000135
	BMI (Obese vs. Non-obese)	0.91	0.78–1.06	0.212	0.593
	Smoking (Smoker vs. Non-smoker)	1.33	1.06–1.69	0.0158	0.0909
*B. breve*	^*^Sex (Male vs. Female)	0.69	0.55–0.86	0.00098	0.00686
	^*^BMI (Obese vs. Non-obese)	0.68	0.55–0.84	0.000283	0.00396
	Smoking (Smoker vs. Non-smoker)	0.66	0.45–0.97	0.0362	0.127
*L. helveticus*	^*^Sex (Male vs. Female)	0.71	0.57–0.88	0.00194	0.00904
	BMI (Obese vs. Non-obese)	0.96	0.78–1.18	0.717	0.921
	Smoking (Smoker vs. Non-smoker)	0.78	0.55–1.12	0.175	0.349

OR: odds ratio; CI_95: 95% confidence interval; *p*_ raw: unadjusted *p*-value; *p*_ FDR: false discovery rate–adjusted *p*-value;

* FDR-adjusted *p* < 0.05.

### 3.3 Effect of probiotic intake on the detection rate of 19 probiotic species

**Figure 4:**
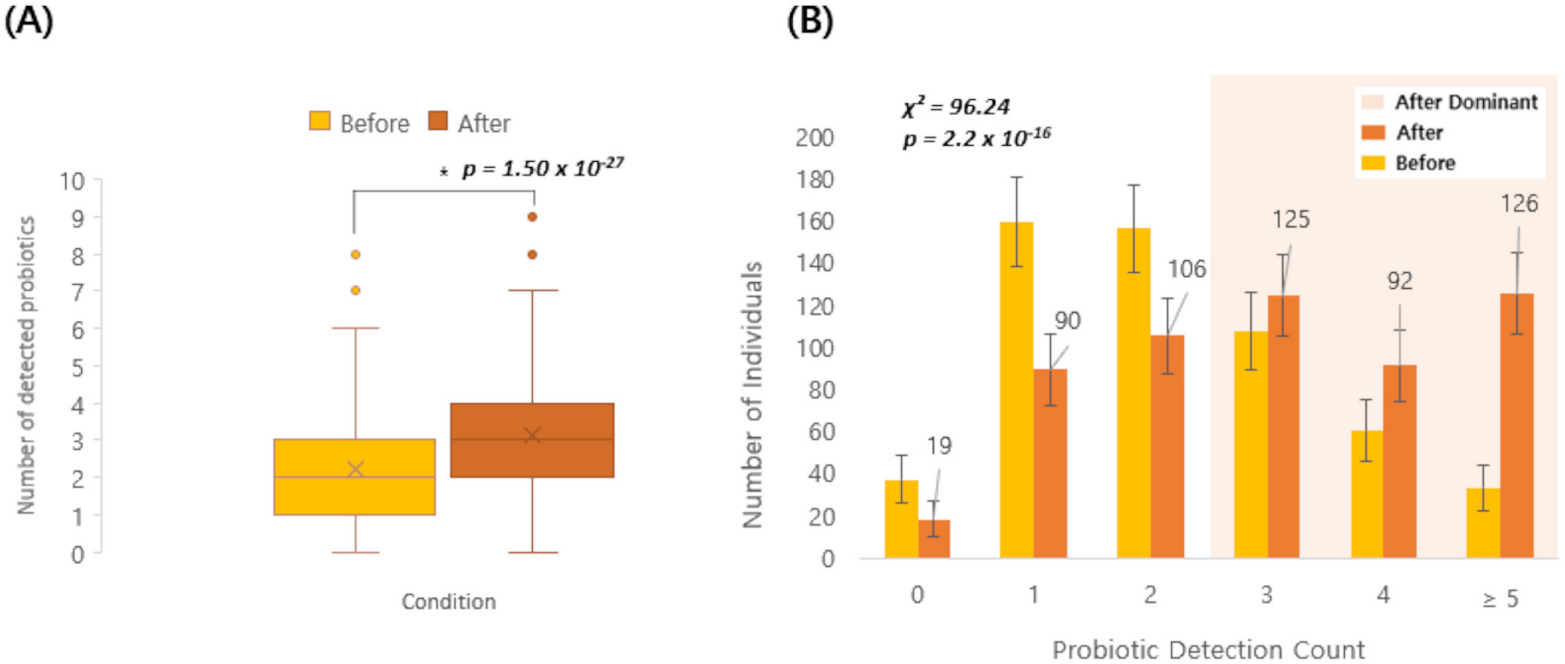
Probiotic detection before and after intake (*n* = 558). **(A)** Box-and-whisker plot showing the number of detected probiotic species (out of 19) before and after probiotic intake in the same individuals. Boxes show the interquartile range (IQR) with the median line; whiskers extend to 1.5 × IQR; points represent individual participants, and “ × ” denotes the mean. Pre–post differences were assessed using a paired, two-sided *t*- test. The mean change (After–Before) was Δmean = 0.95 (95% CI 0.80–1.10) (*p* = 1.50 × 10^−27^). **(B)** Bar plot showing the distribution of individuals by probiotic detection count (0, 1, 2, 3, 4, ≥5) before and after intake. Numbers above bars indicate counts. Error bars represent 95% confidence intervals (*N* = 558; normal approximation). Distributions were compared using a Pearson χ^2^ test (χ^2^ = 96.24, *p* = 2.2 × 10^−16^).

**Figure 5:**
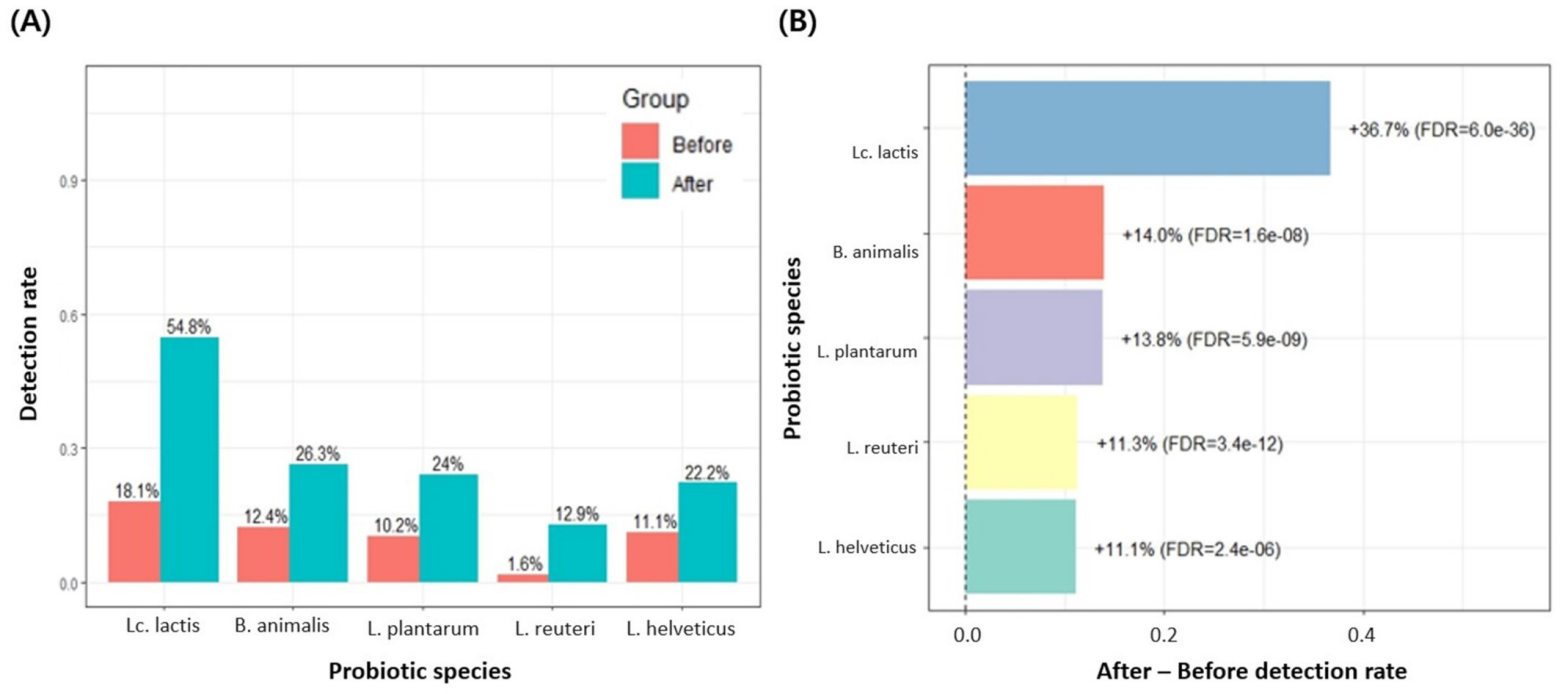
Detection rates of probiotic species with significant changes after intake (FDR < 0.05). **(A)** Among the 19 MFDS-listed probiotic species, only those showing a significant difference in detection rates between the before and after groups (FDR-adjusted *p* < 0.05) are presented. Bars indicate the detection rate (proportion of participants in whom each species was detected) in the before and after groups, and numeric labels above the bars represent the corresponding percentages. *Lc. lactis, B. animalis, L. plantarum, L. reuteri*, and *L. helveticus* all showed significantly higher detection rates after probiotic intake. **(B)** Among the 19 probiotic species, only those with a significant difference between the before and after groups (FDR < 0.05 in Supplementary Figure 1) are shown. The x-axis represents the change in detection rate (After–Before), where positive values indicate an increase after intake. Numerical labels next to each bar show the percent increase and the corresponding FDR-adjusted *p*-value. The vertical dashed line at 0 indicates no change.

Among the 3,450 Korean individuals, a subset of 558 participants underwent gut micro biome testing both before and after probiotic intake. The average number of the 19 probiotic species detected increased from 2.2 before intake to 3.2 after intake, and a paired, two-sided *t*-test confirmed a significant increase (Δmean = 0.95, 95% CI 0.80–1.10; *p* = 1.50 × 10^−27^; Figure 4A). When comparing pre- and post-intake detection rates for each of the 19 probiotic species, several taxa showed significant increases (Supplementary Figure 1). In particular, *Lc. lactis, B. animalis, L. plantarum, L. reuteri*, and *L. helveticus* exhibited marked post-intake increases, with FDR-adjusted *p*-values below 0.05 (Figure 5), and *Lc. lactis* showed the largest change in detection rate (+36.7%, FDR = 6.0 × 10^−36^). Additionally, a higher proportion of individuals had ≥3 detected species after probiotic consumption than before (203/558 [36.4%] vs. 343/558 [61.5%]; Figure 4B). Because 19 species were tested in parallel, we controlled for multiple comparisons using the Benjamini–Hochberg FDR procedure, and only species with FDR < 0.05 were regarded as showing a statistically significant change in detection after intake. No significant differences were observed in alpha diversity indices (Chao1, Shannon, and Faith-pd) between pre- and post-intake samples.

### 3.4 Comparison of alpha diversity and probiotic detection between Korean and Thai individuals

To compare gut micro biome characteristics between Korean and Thai individuals, alpha diversity indices and the number of detected probiotics were analyzed in each group (*n* = 73 per group). As shown in Supplementary Figure 2 and summarized in Supplementary Table 3, phylogenetic diversity was higher in Thai participants than in Korean participants (Faith-pd, *p* < 0.001), and the Shannon index showed a borderline difference with slightly higher values in Thais (*p* = 0.05), whereas Chao1 index did not differ significantly between groups (*p* = 0.73). In contrast, the mean number of detected probiotic species (out of 19 tested taxa) was significantly higher in Korean individuals than in Thai individuals (2.96 ± 1.82 vs. 2.22 ± 1.67; *p* = 0.01).

## 4 Discussion

In this study, we aimed to understand how age, host phenotype, and modifiable lifestyle behaviors, including probiotic intake, are associated with gut micro biota structure in a large Korean cohort. Rather than focusing on a single exposure, we examined age-related differences in microbial diversity and composition, evaluated how demographic and lifestyle factors relate to the detection of specific probiotic taxa, and assessed within-individual changes before and after probiotic use. An exploratory comparison between Korean and Thai urban participants was further included to provide preliminary context for how these patterns may vary across cultural settings. Several important insights were obtained from these analyses.

We observed that gut microbial alpha diversity tended to increase with age, consistent with previous reports in both Western and Asian populations (Odamaki et al., 2016; Xu et al., 2019). All three diversity metrics—Chao1, Shannon, and Faith-pd—were significantly higher in participants aged ≥40 years than in those <40 years, suggesting a gradual increase in microbial diversity across life stages. Age-related shifts in gut microbial diversity likely reflect changes in diet, environmental exposures, and physiological aging processes. Interestingly, Faith-pd showed a slight decline in participants over 70 years of age, which may reflect frailty, polypharmacy, or physiological deterioration associated with advanced age, as reported previously (Yatsunenko et al., 2012). In terms of composition, younger individuals exhibited relatively higher proportions of Bifidobacterium, Bacteroides, and *Blautia*, whereas older individuals had greater relative abundances of *Coprococcus, Prevotella*, and *Faecalibacterium*. These results are consistent with prior observations that taxa abundant in younger populations, such as Bifidobacterium, decrease with age, while fiber-degrading genera, such as *Prevotella*, increase (Odamaki et al., 2016). In contrast, Bacteroides showed a decreasing trend with advancing age in our cohort, whereas studies in other countries have reported different age-related patterns. Such discrepancies are not unexpected, as gut microbial trajectories are strongly shaped by lifestyle, diet, and other non-genetic host factors. Indeed, a study of Korean men and women with a mean age of 50 years also observed an age-related decline in *Bacteroides* alongside an increase in *Prevotella* (Lim et al., 2021), highlighting that population-specific contexts can lead to diverse microbial aging profiles.

In Korea, these age-related microbial differences emerge around the same period in which cardiometabolic risk and lifestyle burden begin to rise. Epidemiological data show that the prevalence of metabolic syndrome and related risk factors increases markedly from the 40s onward, accompanied by unfavorable trends in obesity, leisure-time physical activity, and sedentary behavior in midlife adults (Han et al., 2024; Huh et al., 2021; Kwon et al., 2005). Although we did not directly measure diet or physical activity, the shift from *Bifidobacterium*- and *Bacteroides*-enriched profiles toward greater relative abundances of fiber-associated and saccharolytic genera, such as *Prevotella, Coprococcus*, and *Faecalibacterium*, in individuals ≥40 years may reflect the cumulative impact of long-term dietary patterns and lifestyle changes that characterize Korean midlife (Louis and Flint, 2009; Rivière et al., 2016). At the same time, the coexistence of increased microbial diversity and rising cardiometabolic burden suggests that the higher alpha diversity in this age group does not necessarily correspond to a more favorable metabolic phenotype (Han et al., 2024; Huh et al., 2021; Kwon et al., 2005).

An analysis was conducted on the associations between demographic and lifestyle factors and probiotic detection. Our results showed that women, non-obese individuals, and non-smokers had higher detection rates of three or more probiotic species, suggesting that host demographics and lifestyle characteristics may influence specific aspects of the gut micro biota. To move beyond these univariate comparisons, we additionally fitted multivariable logistic regression models including sex, BMI category, and smoking status. The results indicated that sex and adiposity remained the primary host factors associated with the detection of several species of *Bifidobacterium* and *Lactobacillus* whereas smoking-related effects were comparatively modest and did not persist after multiple-testing correction. It is possible that the marked imbalance in sample size between non-smokers and current smokers in this cohort (3,057 vs. 380 individuals) reduced the precision and statistical power to detect more subtle smoking-related associations; therefore, smoking-related findings should be interpreted with appropriate caution. Moreover, because very low-prevalence probiotic species could not be modeled reliably, these multivariable findings are best interpreted as characterizing host–micro biota relationships among taxa that were at least moderately prevalent in this cohort.

Although no significant differences in alpha diversity were observed across these host variables in our dataset, this does not necessarily imply that demographic and lifestyle factors have no impact. Rather, it is consistent with previous reports showing that factors, such as sex and BMI, can influence the composition and relative abundance of specific microbial taxa without substantially altering overall community diversity (Li et al., 2024). In line with our observation that several species of *Bifidobacterium* and *Lactobacillus* were less frequently detected in men, experimental and clinical work has highlighted that biological sex can shape gut microbiota profiles through differences in sex hormones, diet, and immune function (Rosser et al., 2022). Likewise, the inverse association between obesity and *B. breve* detection is consistent with studies reporting reduced *Bifidobacterium* abundance in individuals with visceral obesity and metabolically adverse phenotypes (Gong et al., 2022), as well as with recent meta-analytic evidence that *Bifidobacterium* supplementation yields small but significant improvements in weight, BMI, and insulin levels among overweight or obese adults (Huang and Cheng, 2025). Taken together, these results suggest that demographic and lifestyle factors may selectively influence the presence of certain taxa, such as probiotics, without causing global restructuring of the gut microbiota.

Here, probiotic supplementation significantly increased the detection of probiotic species in a subgroup of 558 individuals who underwent pre- and post-supplementation analyses. The mean number of detectable probiotic taxa increased from 2.2 to 3.2, and the proportion of individuals with ≥3 detectable taxa rose from 36% to 61% after supplementation. When we compared pre- and post-intake detection rates for each of the 19 probiotic species, five taxa—*Lc. lactis, B. animalis, L. plantarum, L. reuteri*, and *L. helveticus*—showed significant increases. In contrast, alpha diversity indices (Chao1, Shannon, and Faith-pd) did not differ significantly between pre- and post-supplementation samples, indicating that probiotic use mainly altered the detectability of specific taxa rather than reshaping overall community diversity. These findings are consistent with previous studies suggesting that many probiotic strains act as transient residents that interact with the host and resident microbiota to confer health benefits without inducing large-scale, durable restructuring of the gut microbial ecosystem (Ouwehand et al., 2002; Zmora et al., 2018).

The five species that were most responsive to supplementation—*Lc. lactis, B. animalis, L. plantarum, L. reuteri*, and *L. helveticus*—are lactic acid bacteria and bifidobacteria that are widely used as probiotic cultures in fermented dairy products and related functional foods, where they contribute both to fermentation and health-promoting properties (Jena and Choudhury, 2025; Widyastuti et al., 2021). Multiple strains within these genera tolerate acidic and bile conditions, survive gastrointestinal transit, and exert beneficial effects on gut homeostasis and metabolic or systemic outcomes in experimental and clinical settings (Hao et al., 2024; Taverniti and Guglielmetti, 2012), which may partly explain their prominent responsiveness herein. Among them, *Lc. lactis* showed the largest increase in detection, consistent with its role as a key starter culture in cheese and other fermented dairy products and its emerging status as a candidate probiotic, with selected strains demonstrating survival in simulated gastric juice and bile, adhesive or aggregative capacity, and immunomodulatory or anti-pathogenic activities (De Chiara et al., 2024; Kondrotiene et al., 2024). At the same time, our study was not designed to disentangle the relative contributions of dietary exposure vs. intrinsic strain properties. In addition, probiotic use was assessed using a general questionnaire that only classified participants as current users or non-users, without details on specific products, strains, doses, frequency, or duration. Moreover, our outcomes were limited to species-level presence vs. absence in fecal samples. Thus, the observed increases should be interpreted as associations between binary probiotic-use status and species-level detection, rather than as evidence of strain-specific effects, dose–response relationships, or stable long-term colonization.

Finally, an exploratory cross-national comparison revealed modest differences between Korean and Thai participants. Specifically, Thai individuals exhibited higher Faith-pd values, indicating greater overall richness and phylogenetic diversity within their gut micro biota, whereas Chao1 richness and Shannon diversity were broadly similar between the two groups. In contrast, Korean participants demonstrated a higher mean number of detected probiotic species (out of the 19 taxa examined), suggesting that the presence of beneficial taxa may be shaped by host- and culture-specific factors rather than by overall microbial diversity. This finding underscores that a high alpha diversity does not necessarily correspond to a greater abundance or prevalence of specific probiotic species. These differences may be partially attributable to distinct cultural dietary practices (Senghor et al., 2018). Traditional Thai diets are typically rich in a wide variety of plant-based foods and herbs, which provide diverse substrates for microbial fermentation and may support greater microbial diversity. Conversely, the Korean diet is characterized by fermented foods, such as kimchi, soybean paste, and fermented dairy products, which are natural sources of lactic acid bacteria and may contribute to the higher detection rates of probiotic species in this cohort. However, these interpretations remain speculative, as detailed dietary and probiotic intake data were not available for this cross-national subset. Moreover, the Thai samples were collected in Bangkok and therefore represent an urban cohort, and although the Korean participants were recruited nationwide, most regions in Korea are highly urbanized; thus, the comparison primarily reflects differences between urban populations in the two countries. Further, given the relatively small sample size in each group (*n* = 73) and the lack of comprehensive information on residential environment and other potential confounders, these cross-national findings should be regarded as hypothesis-generating rather than definitive and warrant validation in larger, geographically diverse studies. Future studies with larger, demographically well-balanced cohorts from both countries will be needed to more accurately characterize between-country differences in gut micro biota.

Taken together, these analyses suggest a layered view of how the gut micro biota of Korean adults is shaped. Age and the midlife transition around 40 years are primarily reflected in broad shifts in microbial diversity and community composition, whereas demographic and lifestyle factors, such as sex and adiposity appear to exert more selective effects on particular taxa, including species of *Bifidobacterium* and *Lactobacillus*. Probiotic supplementation, in turn, seems to function less as a driver of global diversity and more as a modifier of the presence of targeted probiotic species, especially in individuals who already harbor these taxa at baseline. The exploratory comparison between Korean and Thai urban participants further illustrates that higher alpha diversity does not necessarily coincide with a greater number of probiotic species, highlighting the importance of cultural context, diet, and probiotic use when interpreting micro biome profiles.

While these findings provide important insights, several limitations should be acknowledged. First, most analyses were cross-sectional in nature, restricting our ability to establish causal relationships between host factors and microbial features. Second, probiotic supplementation effects were evaluated only by comparing pre- and post-supplementation samples, without considering dosage, duration, or strain-specific variation. Third, because our study relied primarily on 16S rRNA gene sequencing with the Greengenes 13_8 reference database, fine-scale strain-level diversity and some taxonomic assignments may have been underestimated or less up to date than those obtained with more recent frameworks, such as SILVA or GTDB. We partially mitigated this limitation by complementing the Greengenes-based taxonomy with NCBI RefSeq reference sequences to refine species-level classification where distinguishable by V3–V4 16S rRNA gene sequences. Future investigations incorporating shotgun metagenomic sequencing will be important to validate these findings at higher resolution and to delineate the functional contributions of probiotic and non-probiotic taxa more precisely. Finally, given that the exploratory comparison with the Thai cohort was limited in scope and sample size, the results are presented in the Supplementary material and should be interpreted cautiously.

## 5 Conclusion

This large-scale analysis of 3,450 Korean adults indicates that age, particularly the transition into midlife, is accompanied by broad shifts in gut microbial diversity and taxonomic composition, whereas host factors such as sex, BMI, and smoking status are more selectively associated with the detectability of probiotic-associated taxa. Overall, these findings support a layered ecological model of the gut micro biome in which midlife aging is associated with—or may contribute to—community-wide structural shifts, while host phenotype and lifestyle factors selectively shape which probiotic-associated taxa are detectable. Probiotic use mainly increased the relative abundance/detectability of a subset of probiotic-associated taxa on top of the baseline micro biota without substantially altering alpha diversity, consistent with a transient modulatory effect and supported by the pre–post subgroup showing more frequent detection of several lactic acid bacteria and *Bifidobacterium* species. An exploratory cross-national comparison between Korean and Thai urban participants further showed that higher alpha diversity does not necessarily coincide with a greater number of probiotic species, underscoring the importance of dietary and cultural contexts when interpreting gut micro biota profiles. Collectively, our results refine an ecological view of the Korean gut micro biome by clearly separating broad age-related community shifts from more selective host- and probiotic-associated signals, providing a conceptual framework of the design and interpretation of future population-based micro biome studies.

## Data Availability

The data presented in this study are publicly available. The data can be found here: https://www.ncbi.nlm.nih.gov, accession PRJNA1400113.

## Supplementary material

Supplementary Table 1 List of probiotic species approved by the Ministry of Food and Drug Safety (MFDS), Republic of Korea, and included in this study.

Supplementary Table 2 Multivariable logistic regression for detection of probiotic species.

Supplementary Table 3 Comparison of alpha diversity indices and mean number of detected probiotic species between Korean and Thai participants.

Supplementary Figure 1 Detection rates of probiotic species before and after intake. For each of the 19 MFDS-approved probiotic species, detection rates are shown for the Before and After groups. The y-axis indicates the proportion of participants in whom each species was detected. Asterisks (***) denote species with FDR-adjusted *p*-values < 0.05, < 0.01, and < 0.001, respectively, based on chi-square or Fisher's exact tests with Benjamini-Hochberg correction for multiple comparisons.

Supplementary Figure 2 Comparison of alpha diversity indices and probiotic detection between Korean and Thai individuals (*n* = 73). Box plots comparing gut microbiome alpha diversity and probiotic detection between Korean and Thai individuals. Each box plot displays the median, interquartile range, and outliers; * indicates statistically significant differences (*p* < 0.05) by unpaired *t*-test. Korean and Thai data are shown as blue and green box plots, respectively (left and right boxes in each panel). **(A)** Chao1 index showed no significant difference (*p* = 0.7340). **(B)** Faith's phylogenetic diversity and **(C)** Shannon index were significantly higher in Thai individuals than in Koreans (*p* = 0.0004 and *p* = 0.0482, respectively). **(D)** In contrast, the number of detected probiotics (out of 19 species) was significantly higher in the Korean group (*p* = 0.0120).
