# Supplementary material for: Characterization of the gut micro biota in Koreans and investigation of its association with probiotic consumption: implications for microbial ecology and host health
Source: Front Microbiol. 2026 Jan 30;16:1745533. doi: 10.3389/fmicb.2025.1745533 (PMC12902936; doi:10.3389/fmicb.2025.1745533)
Supplement: Supplementary Table 1 — List of probiotic species approved by the Ministry of Food and Drug Safety (MFDS), Republic of Korea, and included in this study. [file Table_1.docx]

**Supplementary Table 1. List of probiotic species approved by the Ministry of Food and Drug Safety (MFDS), Republic of Korea, and included in this study.**

*MFDS Notification No. 2021-65

| **Genus** | **Species (including subspecies)** |
| --- | --- |
| **Lactobacillus** | *L. acidophilus* *L. gasseri* *L. delbrueckii subsp. bulgaricus* *L. helveticus* |
| **Lacticaseibacillus** | *L. casei*  *L. paracasei* *L. rhamnosus* |
| **Limosilactobacillus** | *L. fermentum* *L. reuteri* |
| **Lactiplantibacillus** | *L. plantarum* |
| **Ligilactobacillus** | *L. salivarius* |
| **Lactococcus** | *L. lactis* |
| **Enterococcus** | *E. faecalis* *E. faecium* |
| **Streptococcus** | *S. thermophilus* |
| **Bifidobacterium** | *B. bifidum* *B. breve* *B. longum* *B. animalis* |
