# Supplementary material for: Characterization of the gut micro biota in Koreans and investigation of its association with probiotic consumption: implications for microbial ecology and host health
Source: Front Microbiol. 2026 Jan 30;16:1745533. doi: 10.3389/fmicb.2025.1745533 (PMC12902936; doi:10.3389/fmicb.2025.1745533)
Supplement: Supplementary Table 2 — Multivariable logistic regression for detection of probiotic species. [file Table_2.docx]

**Supplementary Table 2. Multivariable logistic regression for detection of probiotic species.**

OR, odds ratio; CI_95, 95% confidence interval; p_raw, unadjusted p-value; p_FDR, false discovery rate–adjusted p-value; FDR-adjusted p < 0.05 was considered statistically significant, and predictors with FDR-adjusted p < 0.05 for at least one species are summarized in Table 2.

| **Species** | **Predictor** | **OR** | **CI_95** | **p_raw** | **p_FDR** |
| --- | --- | --- | --- | --- | --- |
| *B.animalis* | Sex (Male vs Female) | 0.79 | 0.65–0.98 | 0.03 | 0.09 |
|  | BMI (Obese vs Non-Obese) | 0.98 | 0.81–1.2 | 0.87 | 0.95 |
|  | Smoking (Smoker vs Non-smoker) | 0.66 | 0.46–0.93 | 0.02 | 0.09 |
| *B.bifidum* | Sex (Male vs Female) | 0.67 | 0.57–0.78 | 0.00 | **0.00** |
|  | BMI (Obese vs Non-Obese) | 0.91 | 0.78–1.06 | 0.21 | 0.59 |
|  | Smoking (Smoker vs Non-smoker) | 1.33 | 1.06–1.69 | 0.02 | 0.09 |
| *B.breve* | Sex (Male vs Female) | 0.69 | 0.55–0.86 | 0.00 | **0.01** |
|  | BMI (Obese vs Non-Obese) | 0.68 | 0.55–0.84 | 0.00 | **0.00** |
|  | Smoking (Smoker vs Non-smoker) | 0.66 | 0.45–0.97 | 0.04 | 0.13 |
| *B.longum* | Sex (Male vs Female) | 0.79 | 0.65–0.96 | 0.02 | 0.06 |
|  | BMI (Obese vs Non-Obese) | 1.05 | 0.87–1.26 | 0.64 | 0.92 |
|  | Smoking (Smoker vs Non-smoker) | 1.12 | 0.84–1.5 | 0.44 | 0.61 |
| *E.faecalis* | Sex (Male vs Female) | 1.13 | 0.86–1.49 | 0.37 | 0.48 |
|  | BMI (Obese vs Non-Obese) | 0.78 | 0.6–1.02 | 0.07 | 0.50 |
|  | Smoking (Smoker vs Non-smoker) | 0.89 | 0.58–1.36 | 0.60 | 0.61 |
| *L.bulgaricus* | Sex (Male vs Female) | 1.18 | 0.79–1.76 | 0.41 | 0.48 |
|  | BMI (Obese vs Non-Obese) | 1.39 | 0.93–2.06 | 0.11 | 0.50 |
|  | Smoking (Smoker vs Non-smoker) | 0.86 | 0.47–1.56 | 0.61 | 0.61 |
| *L.fermentum* | Sex (Male vs Female) | 1.17 | 0.59–2.32 | 0.65 | 0.69 |
|  | BMI (Obese vs Non-Obese) | 1.01 | 0.51–1.98 | 0.98 | 0.98 |
|  | Smoking (Smoker vs Non-smoker) | 0.41 | 0.1–1.73 | 0.22 | 0.39 |
| *L.gasseri* | Sex (Male vs Female) | 0.45 | 0.17–1.19 | 0.11 | 0.23 |
|  | BMI (Obese vs Non-Obese) | 1.86 | 0.77–4.5 | 0.17 | 0.59 |
|  | Smoking (Smoker vs Non-smoker) | 1.46 | 0.41–5.23 | 0.56 | 0.61 |
| *L.helveticus* | Sex (Male vs Female) | 0.71 | 0.57–0.88 | 0.00 | **0.01** |
|  | BMI (Obese vs Non-Obese) | 0.96 | 0.78–1.18 | 0.72 | 0.92 |
|  | Smoking (Smoker vs Non-smoker) | 0.78 | 0.55–1.12 | 0.18 | 0.35 |
| *L.plantarum* | Sex (Male vs Female) | 0.86 | 0.69–1.08 | 0.19 | 0.30 |
|  | BMI (Obese vs Non-Obese) | 1.02 | 0.82–1.26 | 0.88 | 0.95 |
|  | Smoking (Smoker vs Non-smoker) | 0.86 | 0.6–1.22 | 0.39 | 0.61 |
| *L.reuteri* | Sex (Male vs Female) | 0.7 | 0.44–1.12 | 0.14 | 0.24 |
|  | BMI (Obese vs Non-Obese) | 0.87 | 0.56–1.35 | 0.53 | 0.92 |
|  | Smoking (Smoker vs Non-smoker) | 1.25 | 0.65–2.43 | 0.51 | 0.61 |
| *L.salivarius* | Sex (Male vs Female) | 1.37 | 0.8–2.33 | 0.25 | 0.35 |
|  | BMI (Obese vs Non-Obese) | 0.9 | 0.54–1.52 | 0.70 | 0.92 |
|  | Smoking (Smoker vs Non-smoker) | 1.76 | 0.92–3.34 | 0.09 | 0.20 |
| *Lc.lactis* | Sex (Male vs Female) | 0.96 | 0.81–1.15 | 0.69 | 0.69 |
|  | BMI (Obese vs Non-Obese) | 1.06 | 0.89–1.26 | 0.52 | 0.92 |
|  | Smoking (Smoker vs Non-smoker) | 0.69 | 0.52–0.93 | 0.01 | 0.09 |
| *S.thermophilus* | Sex (Male vs Female) | 0.88 | 0.75–1.03 | 0.12 | 0.23 |
|  | BMI (Obese vs Non-Obese) | 1.03 | 0.88–1.2 | 0.72 | 0.92 |
|  | Smoking (Smoker vs Non-smoker) | 0.79 | 0.62–1.02 | 0.07 | 0.19 |
