# Supplementary material for: Characterization of the gut micro biota in Koreans and investigation of its association with probiotic consumption: implications for microbial ecology and host health
Source: Front Microbiol. 2026 Jan 30;16:1745533. doi: 10.3389/fmicb.2025.1745533 (PMC12902936; doi:10.3389/fmicb.2025.1745533)
Supplement: Supplementary Table 3 — Comparison of alpha diversity indices and mean number of detected probiotic species between Korean and Thai participants. [file Table_3.docx]

**Supplementary Table 3. Comparison of alpha diversity indices and mean number of detected probiotic species between Korean and Thai participants.**

*p < 0.05 for differences between Korean and Thai participants (Welch’s t-test). Avg. probiotic spp, average number of detected probiotic species out of 19 MFDS-listed probiotic taxa.

| **Metric** | **Korea (mean ± SD)** | **Thailand  (mean ± SD)** | **Mean difference  (KOR -THA) (95% CI)** | **p-value** |
| --- | --- | --- | --- | --- |
| Chao1 | 237.32 ± 108.74 | 242.61 ± 75.94 | -5.3 (-36.0 to 25.4) | 0.73 |
| Faith-pd* | 14.35 ± 4.08 | 16.59 ± 3.41 | -2.2 (-3.5 to -1.0) | 0.00 |
| Shannon | 5.71 ± 0.71 | 5.94 ± 0.66 | -0.2 (-0.4 to 0.0) | 0.05 |
| Avg. probiotic spp* | 2.96 ± 1.82 | 2.22 ± 1.67 | 0.7 (0.2 to 1.3) | 0.01 |
