# Supplementary figures and images for: Characterization of the gut micro biota in Koreans and investigation of its association with probiotic consumption: implications for microbial ecology and host health
Source: Front Microbiol. 2026 Jan 30;16:1745533. doi: 10.3389/fmicb.2025.1745533 (PMC12902936; doi:10.3389/fmicb.2025.1745533)

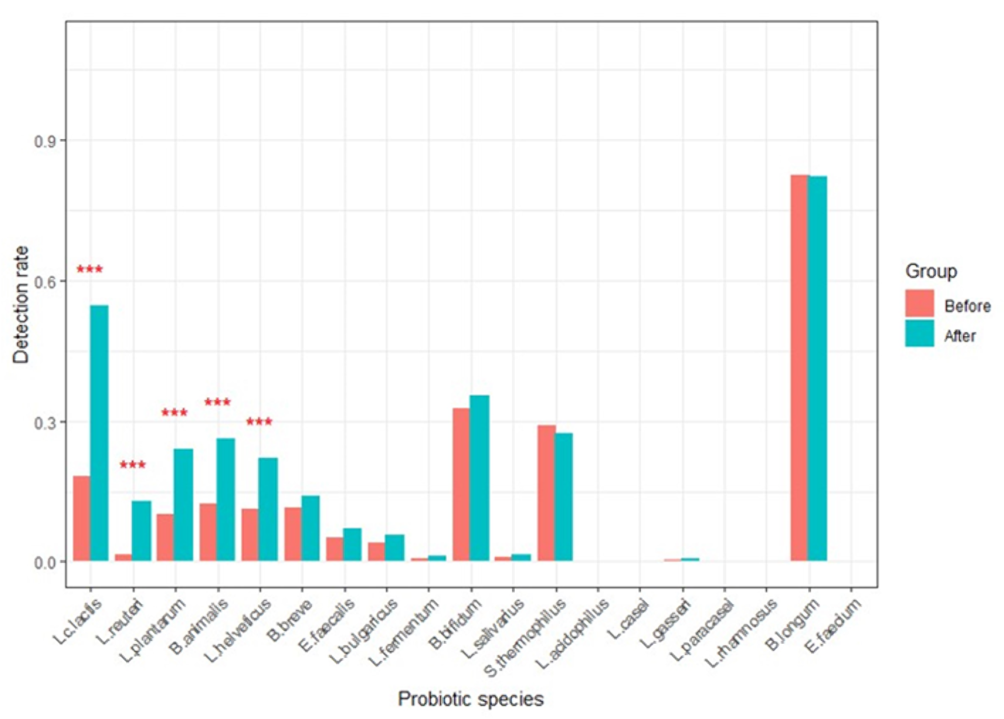

Supplement: Supplementary Figure 1 — Detection rates of probiotic species before and after intake. For each of the 19 MFDS-approved probiotic species, detection rates are shown for the Before and After groups. The y-axis indicates the proportion of participants in whom each species was detected. Asterisks (***) denote species with FDR-adjusted p-values < 0.05, < 0.01, and < 0.001, respectively, based on chi-square or Fisher's exact tests with Benjamini-Hochberg correction for multiple comparisons. [file Image_1.tiff]

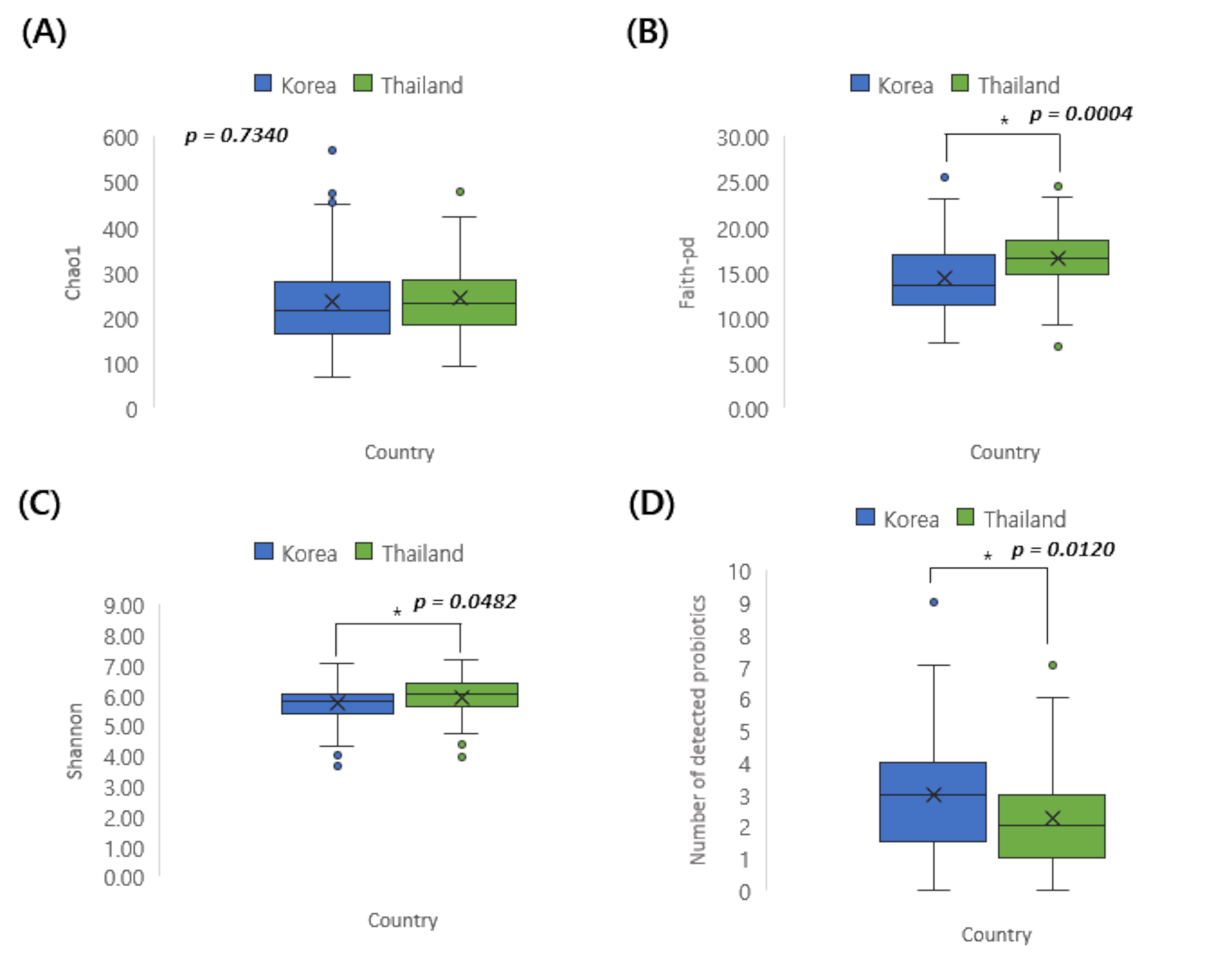

Supplement: Supplementary Figure 2 — Comparison of alpha diversity indices and probiotic detection between Korean and Thai individuals (n = 73). Box plots comparing gut microbiome alpha diversity and probiotic detection between Korean and Thai individuals. Each box plot displays the median, interquartile range, and outliers; * indicates statistically significant differences (p < 0.05) by unpaired t-test. Korean and Thai data are shown as blue and green box plots, respectively (left and right boxes in each panel). (A) Chao1 index showed no significant difference (p = 0.7340). (B) Faith's phylogenetic diversity and (C) Shannon index were significantly higher in Thai individuals than in Koreans (p = 0.0004 and p = 0.0482, respectively). (D) In contrast, the number of detected probiotics (out of 19 species) was significantly higher in the Korean group (p = 0.0120). [file Image_2.tiff]
